# Design of an Underwater Treadmill System for rehabilitation of older obese adults: a pre-post study

**DOI:** 10.1186/s12877-019-1334-5

**Published:** 2019-11-14

**Authors:** C. Kittichaikarn, V. Kuptniratsaikul

**Affiliations:** 1 Chawalit Kittichaikarn Department of Mechanical Engineering, Faculty of Engineering, Kasetsart University, 50 Ngam Wong Wan Road, Jatujak, Bangkok, 10900 Thailand; 2 Vilai Kuptniratsaikul Department of Rehabilitation Medicine, Faculty of Medicine Siriraj Hospital, Mahidol University, 2 Wanglang Road, Bangkoknoi, Bangkok, 10700 Thailand

**Keywords:** Underwater treadmill, Quadriceps strength, Pain, Obesity, Knee osteoarthritis

## Abstract

**Background:**

Patients with knee osteoarthritis (OA) who are obese have problems performing land-based exercises. The reduced joint stress associated with aquatic exercise may benefit these patients. This study aimed to develop an underwater treadmill (UTM) machine that is affordable and suitable for use in developing countries, and to evaluate its efficacy in decreasing pain and increasing functional improvement.

**Methods:**

Clinical testing of the UTM machine was performed in an outpatient setting at Siriraj Hospital during January–June 2017. Patients with knee OA, aged 50–85 years, numerical rating scale (NRS) ≥5/10, and body mass index (BMI) ≥25 kg/m ^2^ were recruited. The UTM exercise protocol was 30 min/session, 3 days/week, for 4 weeks. The main outcomes were NRS pain score, 6-min walk distance (6MWD), quadriceps strength (QS) and body weight. Those outcomes were evaluated at baseline and at week 4.

**Results:**

The UTM was constructed with safety, ergonomically designed and user-friendly control panel with push button for emergency stopping. Thirty patients were included for clinical testing. The mean age was 62.8 years, and almost all were female. The mean BMI was 28.9 kg/m ^2^. Most patients (65.0%) developed bilateral knee OA, used pain medication (56.7%), and engaged in regular knee exercise (73.3%). Of the 30 enrolled patients, 6 withdrew. All of the remaining 24 patients attended all 12 sessions. The mean difference between baseline and the end of the study was − 2.3 (95% CI: − 3.0, − 1.5; *p* < 0.001) for NRS pain; 34.9 m (95% CI: 14.1, 55.8; *p* = 0.002) for 6MWD; and, 1.8 kg (95% CI, 1.1, 2.6; *p* < 0.001) for QS. Concerning adverse events, 4 patients (15.4%) developed muscle pain, 2 patients (7.7%) had joint pain, and 1 patient (3.9%) withdrew due to severe knee pain. Two-thirds of patients described themselves as being ‘very satisfied’ with UTM exercise, and approximately 90% of participants rated their symptoms as ‘improved’ or ‘much improved’.

**Conclusions:**

4-week exercise with UTM can significantly improve NRS pain, 6MWD, and QS. UTM could be an alternative treatment for patients with knee OA who are obese due to small size, durability, and ecofriendly design as an exercise modality.

## Background

Knee osteoarthritis (OA) is the most common degenerative disease of the musculoskeletal system worldwide. Overweight and obese older adults are the most likely subpopulation to develop knee OA. The prevalence of knee OA among older adults living in the community in Thailand was reported to be 34.5–45.6% [1]. Knee OA adversely affects activities of daily living, including walking, going up and down stairs, and transferring, due to excessive stress on the knee joint, especially in overweight and obese patients. Knee exercise is recommended by all knee OA guidelines [2–5]; however, patients with knee OA who are obese have problems performing land-based exercises.

Many studies have investigated the effect of aquatic exercise. Roper, et al. studied the effect of aquatic treadmill exercise in 14 patients with knee OA. That study had a cross-over design that compared between aquatic and land treadmill exercise for 1 week (3 sessions). They found that aquatic treadmill exercise improved angular velocity and decreased pain compared to land treadmill exercise [6]. Mackay-Lyons conducted a randomized controlled trial (RCT) of land treadmill aerobic training (3 times/week) for 3 months at 60–80% maximum heart rate in stroke patients, and improvement in both cardiovascular fitness and gait was observed [7]. Some studies compared the effect of aquatic treadmill versus that of land treadmill on cardiorespiratory response [8–10], while another study investigated the effect of exercise on blood lipid and lipoprotein profiles [11]. In contrast, we set forth to learn more about the effect of exercise on pain and functional improvement.

In 1996, Mangione, et al. suggested using a land treadmill with body weight support in older adults with OA, but this type of exercise/therapy could not relieve pain [12]. Aquatic treadmill exercise may be an alternative treatment in this patient population. The buoyancy effect in water can reduce loads on painful joints and can improve the flexibility and stability of muscles, both of which may give a patient more confidence to walk. In 2009, Greene, et al. studied the efficacy of underwater treadmill (UTM) compared to land-based treadmill (LTM) for 12 weeks in 57 overweight or obese adults (36 sessions). They found that both types of exercise could improve fitness and body composition [13]. However, physicians should be aware of the reported potential adverse effects that can result from land-based exercise [14]. UTM-based exercise and therapy is not yet widely available in Thailand due to its high cost, and the fact that these systems need to be imported from abroad. Accordingly, the aim of this study was to develop an underwater treadmill (UTM) system that could be used to rehabilitate and/or improve the fitness of older patients with knee OA who are obese, and to evaluate the efficacy of this system relative to decreasing pain and increasing functional improvement.

## Methods

The aim of this project was to design, develop, and clinically test a UTM that is both affordable and suitable for use in developing countries. UTM development-related information is presented in the supplementary file. The UTM was developed and assembled at the Department of Mechanical Engineering, Faculty of Engineering, Kasetsart University, Bangkok, Thailand, and it was installed at the Department of Rehabilitation Medicine, Faculty of Medicine Siriraj Hospital, Mahidol University, Bangkok, Thailand. Clinical testing of the UTM machine was performed in an outpatient setting during January to June 2017. This study was conducted in accordance with the ethical principles of the Declaration of Helsinki. This study was approved by the Institutional Review Board of Faculty of Medicine Siriraj Hospital, Mahidol University, Bangkok, Thailand (COA no. Si 185/2013). All recruited participants provided written informed consent.

Participants were recruited via publicly displayed printed posters that introduced and described the study. Participants with knee OA who satisfied all of the inclusion criteria and none of the exclusion criteria were recruited. The inclusion criteria were patients with knee OA, aged 50–85 years, numerical rating scale (NRS) ≥5 (range: 0–10), and body mass index of Asian obese population (BMI ≥25 kg/m^2^) [15]. Patients with bowel or bladder incontinence, skin ulcer, and inability to walk due to severe medical problems were excluded. The UTM exercise protocol for this study was 30 min per session, 3 days per week, for 4-week duration. Regarding intensity, the therapist titrated the walking speed from 0.4 up to 0.8 m/second, depending on patient capability.

The primary outcome was NRS pain score, and the secondary outcomes included 6-min walking distance (6MWD), body weight, and quadriceps strength. NRS pain score ranges from 0 to 10, with a higher score representing a higher level of pain intensity [16]. 6MWD is the distance that a patient can walk as fast as he/she can in 6 min [17]. Quadriceps strength in the dominant leg was assessed twice in a sitting position using a hand-held dynamometer [Lafayette Manual Muscle Test System (MMT®) model 01163; Lafayette Instruments, Lafayette, IN, USA]. Each participant was asked to extend his/her leg against resistance applied at the lower part of leg by one physical therapist [18]. The maximal value was chosen. In case of bilateral knee pain, quadriceps strength was measured on the weaker side. All outcomes were evaluated twice - at baseline and at the end of week 4. At the end of the study, adverse events (AEs) that occurred during UTM use were recorded, including muscle pain, joint pain, falling, swelling, and fatigue. Patient satisfaction was evaluated as unsatisfied, satisfied, or very satisfied with UTM exercise. Patient global assessment was rated at the end of the study as improved, much improved, or no observable change.

### Statistical analysis

The sample size for this study was calculated using NRS pain score from a study by Dias, et al. [19], with 90% power and a significance level of 5%. The pain scores at week 0 and week 6 in that study were 51.0 (SD 20.4) and 37.7 (SD 16.5). Using NQuery Sample Size Software (Statistical Solutions Ltd., Cork, Ireland), the calculated minimum sample size for this pre-post study design was 27 patients. We then increased that number to compensate for an estimated dropout rate for any cause of 10%. The resulting sample size was 30 patients. The minimal clinically important difference (MCID) used was 2.0 for NRS pain score [20], and 79 m for 6MWD [21].

Baseline demographic, anthropometric, and clinical characteristics of study participants are presented as mean ± standard deviation (normal distribution) or median and range (non-normal distribution) for continuous data, and as number and percentage for categorical data. Paired *t*-test was used to analyze the mean differences between baseline and end of the study for primary and secondary outcomes with normally distributed data. Per-protocol analysis was used to analyze the outcomes. A *p*-value of less than 0.05 was considered statistically significant for all tests. SPSS Statistics (SPSS, Inc., Chicago, IL, USA) was used to perform all statistical analyses.

## Results

**Fig. 1:**
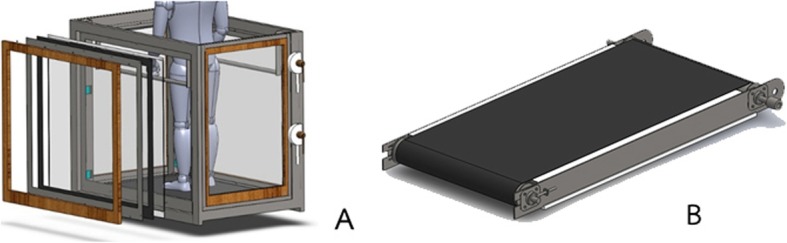
The UTM specification and design characteristics

**Fig. 2:**
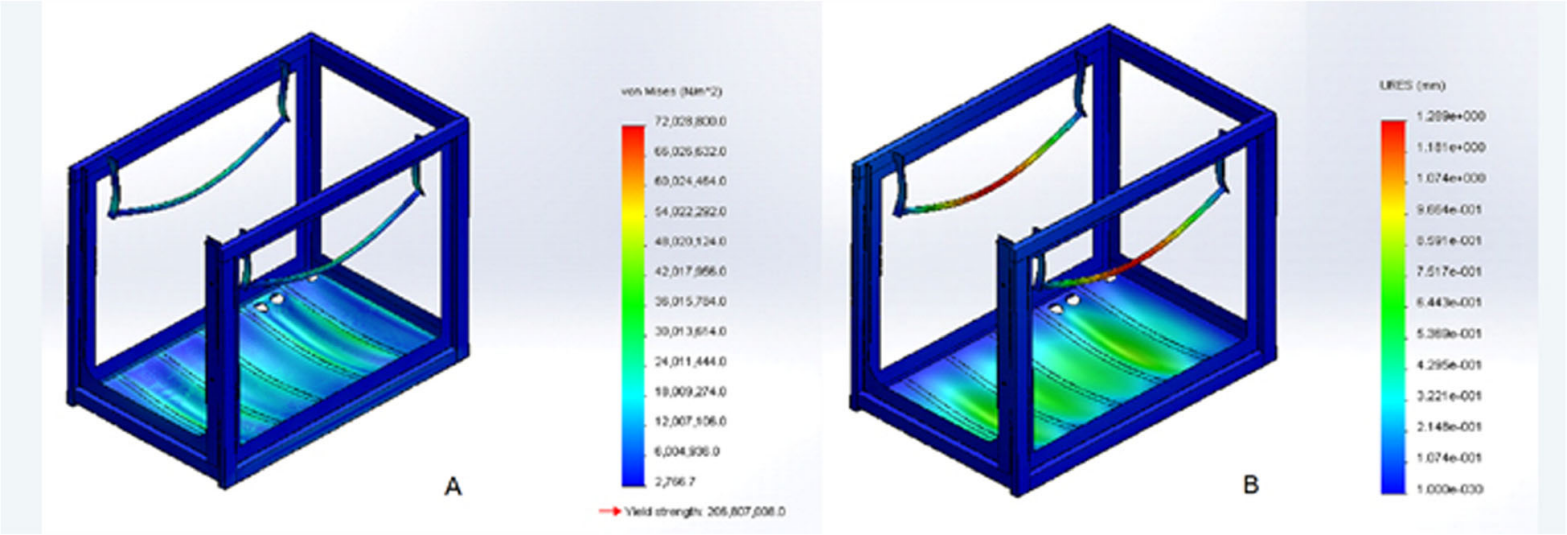
Stress analyses using the finite element method

**Fig. 3:**
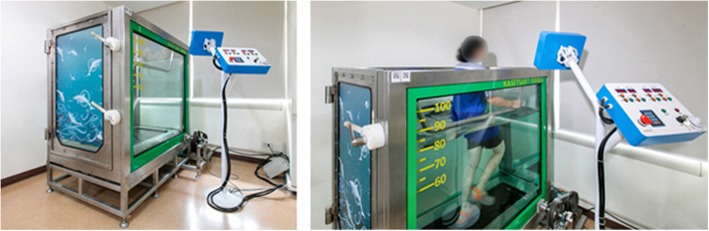
The fully assembled UTM

The design and construction details of the UTM machine are presented in the supplementary file, and the UTM specification and design characteristics are shown in Fig. 1a and Fig. 1b. Stress analyses using the finite element method are shown in Fig. 2a and Fig. 2b. The fully assembled UTM is shown in Fig. 3a and Fig. 3b.

**Fig. 4:**
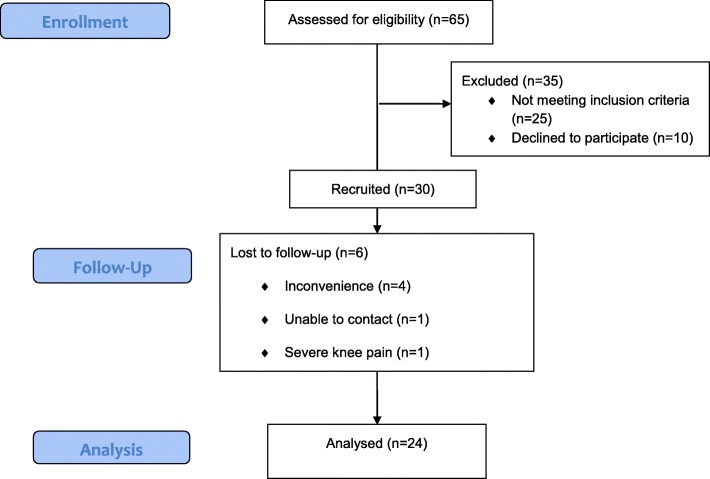
Flow diagram of the study protocol

To assess clinical results, thirty obese knee OA patients were enrolled in this study. Demographic, anthropometric, and clinical characteristics of the study population are shown in Table 1. The mean age of patients was 62.8 years, and almost all patients were female. The mean BMI was 28.9 kg/m^2^. Two-thirds of patients had an education level below bachelor’s degree. More than half of patients (65.0%) developed bilateral knee OA, used pain medication (56.7%), and engaged in regular knee exercise (73.3%). Some used knee support (40.0%), and a few used gait aids (13.3%). Of the 30 enrolled patients, 6 (20%) withdrew from the study due to inconvenience (*n* = 4), lost contact (*n* = 1), and severe knee pain (*n* = 1) (Fig. 4). Regarding participant compliance with the study exercise protocol, all 24 of the patients that completed the study attended all 12 of the scheduled UTM exercise sessions. Among the 6 patients who withdrew from the study, the number of exercise sessions attended ranged from 0 to 10.

**Table 1:** Baseline demographic, anthropometric, and clinical characteristics of study participants

Characteristics	(*n* = 30)
Age (yrs), mean ± SD	62.8 ± 6.9
Female gender, n (%)	29 (96.7%)
BMI (kg/m^2^), mean ± SD	28.9 ± 3.5
Onset of disease (yrs), median (range)	2.0 (0.1, 30.0)
Affected side: both, n (%)	26 (65.0%)
Using gait aids, n (%)	4 (13.3%)
Using knee support, n (%)	12 (40.0%)
Using pain medication, n (%)	17 (56.7%)
Regular knee exercise, n (%)	22 (73.3%)
Pain (NRS; 0–10), mean ± SD	6.4 ± 1.3

Mean NRS pain score, body weight, 6MWD, and quadriceps strength at week 0 and week 4, and the mean difference between pre- and post-treatment using per-protocol analysis are shown in Table 2. Per-protocol analysis revealed a statistically significant difference between pre- and post-treatment for NRS pain, 6MWD, and quadriceps strength, but not for body weight. The mean difference between baseline and the end of the study was − 2.3 (95% CI: − 3.0, − 1.5; *p* < 0.001) for NRS pain; 34.9 m (95% CI: 14.1, 55.8; *p* = 0.002) for 6MWD; and, 1.8 kg (95% CI: 1.1, 2.6; *p* < 0.001) for quadriceps strength.

**Table 2:** Mean NRS pain, body weight, 6MWD, and quadriceps strength at week 0 and week 4, and mean difference between pre- and post-treatment (per-protocol analysis, *n* = 24)

Parameters	Week 0 mean ± SD	Week 4 mean ± SD	Mean difference^#^ (95% CI)	*p*-value
NRS (0–10)	6.4 ± 1.2	4.1 ± 2.0	−2.3 (−3.0, −1.5)	*< 0.001*
Body weight (kg)	70.5 ± 9.8	70.3 ± 9.9	−0.2 (−0.8, 0.5)	0.697
6MWD (meters)	294.4 ± 84.0	329.3 ± 73.1	34.9 (14.1, 55.8)	*0.002*
QS (kg)	8.4 ± 3.1	10.2 ± 3.3	1.8 (1.1, 2.6)	*< 0.001*

A *p*-value< 0.05 indicates statistical significance

^#^Mean difference = week 4 – week 0; using paired *t*-test

Abbreviations: *NRS* numerical rating scale, *6MWD* 6-min walk distance; *QS*, quadriceps strength, *SD* standard deviation, *CI* confidence interval

Concerning adverse events (AE) that occurred after recruitment (including patients lost due to AE), 4 patients (15.4%) developed muscle pain, 2 (7.7%) had joint pain, and 1 (3.9%) was lost to follow-up due to severe knee pain. No study participants reported falling, joint swelling, or fatigue (Table 3). Two-thirds of patients described being ‘very satisfied’ with UTM, and approximately 90% of participants rated their symptoms as either ‘improved’ or ‘much improved’ (Table 4).

**Table 3:** Adverse events of treatment in older obese knee osteoarthritis (OA) patients (*n* = 26)

Adverse events (AE)	n (%)
Muscle pain	4 (15.4%)
Joint pain	2 (7.7%)
Severe knee pain	1 (3.9%)
Falling	0 (0.0%)
Joint swelling	0 (0.0%)
Fatigue	0 (0.0%)

AE included all events that occurred after recruitment, including those that occurred among patients who had to withdraw from the study due to AE

**Table 4:** Patient satisfaction and patient global assessment after treatment at week 4 (*n* = 24)

Assessment parameter	n (%)
Patient satisfaction	
Very satisfied	16 (66.7%)
Satisfied	7 (29.2%)
Unsatisfied	1 (4.1%)
Patient global assessment	
Much improved	9 (37.5%)
Improved	12 (50.0%)
No observable change	3 (12.5%)

## Discussion

Despite the fact that aerobic exercise has been proven beneficial relative to strength and aerobic fitness in patients with knee OA, land-based exercise is associated with musculoskeletal risk due to high impact force on the knee joint [14]. Aquatic exercise may be an alternative in patients with knee OA who are obese that cannot perform land-based exercise due to knee pain. The buoyancy effect of water may reduce stress on lower extremity joints [13]. The results of this study revealed that UTM could reduce pain, improve quadriceps strength, and increase the distance walked in 6 min, but that it could not reduce body weight. The observed reduction in pain may be the result of improved quadriceps strength. A previous study reported that treadmill walking could modulate inflammatory cytokines, including tumor necrosis factor-alpha (TNF-α), interleukin-2 (IL-2), interleukin-4 (IL-4), and interleukin-6 (IL-6) [22]. Reduction of these cytokines may delay the progression of osteoarthritis. The American College of Sports Medicine (ACSM) recommends non-weight bearing exercise for fitness in older adult patients with knee OA who are overweight and obese [23].

Concerning the minimal clinically important difference (MCID) of our findings, we found that UTM therapy could reduce the mean NRS pain score (mean difference: 2.3), which was a greater reduction than that reported by Farrar, et al. (mean difference: 2.0) [20]. However, the mean difference in the 6MWD in our study was less than the MCID reported by Naylor, et al. (35 m vs. 79 m, respectively) [21] who studied OA patients awaiting arthroplasty. Even though the walking distance in our study improved after treatment, the improvement was not clinically significant. In addition, use of the UTM was unable to effectuate reduction in body weight. Excessive caloric intake and a lack of physical activity due to knee pain are the most likely causes of weight gain in this population. Moreover, twelve 30-min sessions exercising on the UTM may not be sufficient to promote weight reduction and/or to increase walking distance. Accordingly, an extended duration exercise program coupled with diet control should be considered.

Regarding complaints of musculoskeletal pain, 15.4% of patients reported muscle pain, and 7.7% of patients described having joint pain. These adverse events may be due to inadequate warm-up, and/or to excessive muscle stress. Participants have to exercise against water resistance during continuous walking, and one-fourth of our patients did not exercise regularly before entering the study. Therefore, therapists should emphasize the importance of warm-up before commencing UTM exercise.

The aim of this study was to assess the applicability and efficacy of the UTM, which was developed by our team. A sample size of 30 patients was calculated based on previous study [19]. Since our participants were voluntarily recruited via publicly displayed printed posters, they might be assumed to be motivated volunteers that could exhibit more interest and cooperation than similarly affected patients within the general population. However, 6 (20%) of our patients were lost to follow-up. Of those, 4 patients withdrew due to inconvenience to continue and complete the study. Disability- and transportation-related challenges made it difficult for some patients to comply with the treatment schedule. Future studies should consider these factors in their study design and sample size calculation. Additionally, the resulting small sample size in this study may have introduced selection bias that may have adversely influenced the results.

This study has some mentionable limitations. First, the non-randomized design of our study renders it vulnerable to selection bias. In addition, the grade of evidence was considered low in this study due to its pre-post design. However, this study design was adopted to facilitate the collection of preliminary data prior to testing using a randomized controlled design. Second, due to reasons that included transportation availability, travel distance, disability-related challenges, and frequency of hospital visits per week, 20% of our study population withdrew from this study. This substantial reduction in sample size may have introduced selection bias, and may have given our study insufficient statistical power to identify all significant differences and associations. Third, most participants in this study were female, and this may limit the generalizability of our findings to males. This difference between males and females in our study may be due to a higher reported prevalence of knee OA in females than in males (39.8% vs. 22.6% respectively) [24]. Fourth, after the unanticipated withdrawal of 20% of our study population, our study’s sample size fell below the calculated minimum sample size requirement. As such, it is unclear whether or not our results should be considered generalizable to a large group of patients suffering from OA. Fifth and last, the UTM is owned by two collaborating universities, and this analysis was conducted by the people who designed, developed, and clinically tested the UTM. Therefore, external evaluation to confirm the findings of this study is warranted. Future study should also include evaluation of more dimensions of functional status, ADL status, community mobility, and quality of life.

The author-designed UTM profiled in this study was given the name “*Aquatread*”. It is a low-cost, small footprint, ecofriendly therapeutic/exercise modality that can provide aquatic exercise to older obese patients with knee OA via continuous treadmill walking. Since its installation over 2 years ago, *Aquatread* is still functioning fully to its design and performance specifications. With regard to user satisfaction, most participants described being satisfied to very satisfied, and their global improvement rating increased from improved to much improved after beginning use of the *Aquatread*. Taken together, these findings may support the benefit of underwater treadmill for treatment of patients with knee OA who are obese. As recommended by the study of Bartels, et al., aquatic exercise could be used during the initial phase of exercise program [25].

## Conclusion

The UTM profiled in this report was designed, fabricated, and clinically tested by the authors of this study. Of the 4 measured parameters, NRS pain, 6MWD, and quadriceps strength were all significantly improved at the end of the study. However, *Aquatread* therapy had no significant effect on body weight. Although adverse events were reported by 7 patients (1 of those withdrew from the study due to severe knee pain), two-thirds of patients described themselves as being ‘very satisfied’ with *Aquatread*, and approximately 90% of participants rated their symptoms as ‘improved’ or ‘much improved’. After 2 years of use, *Aquatread* continues to function reliably and deliver according to its designed performance specifications.

## Data Availability

The datasets generated and/or analyzed during the current study are available from the corresponding author upon reasonable request.

## Abbreviations

6MWD: 6-min walking distance
ACSM: American College of Sports Medicine Abbreviations
AE: Adverse event
BMI: Body mass index
CAD: Computer-aided design
IL: Interleukin
LTM: Land-based treadmill
NRS: Numerical rating scale
OA: Osteoarthritis
RCT: Randomized controlled trial
SD: Standard deviation
SIRB: Siriraj Institutional Review Board
TNF-α: Tumor necrosis factor-alpha
UTM: Underwater treadmill
